# High-throughput precise particle transport at single-particle resolution in a three-dimensional magnetic field for highly sensitive bio-detection

**DOI:** 10.1038/s41598-022-10122-1

**Published:** 2022-04-16

**Authors:** Roozbeh Abedini-Nassab, Reza Shourabi

**Affiliations:** 1 Faculty of Mechanical Engineering, Tarbiat Modares University, P.O. Box: 14115-111, Tehran, Iran; 2 Department of Electrical Engineering, University of Neyshabur, Neyshabur, Iran

**Keywords:** Assay systems, Biomedical engineering

## Abstract

Precise manipulation of microparticles have fundamental applications in the fields of lab-on-a-chip and biomedical engineering. Here, for the first time, we propose a fully operational microfluidic chip equipped with thin magnetic films composed of straight tracks and bends which precisely transports numerous single-particles in the size range of ~ 2.8–20 µm simultaneously, to certain points, synced with the general external three-axial magnetic field. The uniqueness of this design arises from the introduced vertical bias field that provides a repulsion force between the particles and prevents unwanted particle cluster formation, which is a challenge in devices operating in two-dimensional fields. Furthermore, the chip operates as an accurate sensor and detects low levels of proteins and DNA fragments, being captured by the ligand-functionalized magnetic beads, while lowering the background noise by excluding the unwanted bead pairs seen in the previous works. The image-processing detection method in this work allows detection at the single-pair resolution, increasing the sensitivity. The proposed device offers high-throughput particle transport and ultra-sensitive bio-detection in a highly parallel manner at single-particle resolution. It can also operate as a robust single-cell analysis platform for manipulating magnetized single-cells and assembling them in large arrays, with important applications in biology.

## Introduction

Recently, there has been considerable interest in the manipulation of micro-particles (e.g., microbeads and cells) in a microfluidic environment at single-particle resolution. The precise positioning of various cell types has crucial applications in tissue and organ engineering^1,2^. Moreover, transporting beads carrying biological entities or single living cells play a key role in the field of single-cell analysis (SCA)^3–7^ and bio-detection^8–11^, where a large amount of wealthy data is exported. In diseases such as cancer or infectious diseases such as the one caused by human immunodeficiency virus (HIV), rare cells in highly heterogeneous cell populations in tissues (e.g., tumors) or biofluids (e.g., blood) define the patient destiny. Studying the heterogeneity buried among these cell populations is ignored with the traditional bulk level methods, and understanding the underlying phenomena at the single-cell level needs novel SCA tools. Although the available SCA methods have already resulted in interesting achievements^12–17^, the field is in its infancy, and more advanced tools are needed.

To date, various particle transport methods based on optical^18–21^, acoustic^22–26^, electric^27,28^, and magnetic^29–33^ forces are introduced. Optical manipulation provides precise control on single particles; however, its slow nature (i.e., limited number of particles to be manipulated, simultaneously) limits its throughput^20^. Also, this method may suffer from limitations such as cell viability concerns, lack of selectivity, optical interferences, and so on^18,34–36^. Acoustic-based techniques are shown to be appropriate label-free methods for manipulating particles; however, access to single particles is challenging. The number of particles in the single-particle transport methods based on electric forces is normally limited. These methods (e.g., dielectrophoresis or electrowetting) sometimes operate at high voltages. Thus, a robust high-throughput method for precise on-chip transport of particles at single-particle resolution in a highly parallel fashion is needed.

Magnetic-based particle manipulation is considered a method for remotely sorting, separating, and transporting magnetic particles^31,37,38^. In the techniques where external permanent magnets or coils control the magnetic particles^38,39^, precise control over single particles is not seen. This control can be achieved in the devices based on embedded micro-coils^40–42^, where particles are manipulated by selectively switching on/off the micro-coils along the trajectory path. But the wiring system in these chips may become complicated, and the current-carrying coils may generate problematic heat if the number of coil windings is insufficient.

Applying an appropriate external magnetic field to properly designed magnetic thin films embedded in the lab-on-a-chip systems provides appropriate energy distribution for manipulating magnetic particles^43–45^. In our previous works, we have introduced a new class of magnetomicrofluidic chips, called magnetophoretic circuits, to precisely manipulate the magnetic micro-particles^46–51^. In these circuits, all magnetic particles (e.g., tens of thousands of particles) are synced with the external rotating magnetic field and move along magnetic circuits in a controlled fashion. The velocity of the particles is proportional to the frequency of the external magnetic field, which resembles Ohm’s law in the electrical circuit, where the electron current is proportional to the applied electric potential. In addition to conductors, we have also introduced other circuit elements such as diodes, capacitors, and transistors in analogy to the electrical circuits. By combining these circuit elements, we designed a random-access memory (RAM) in which we “write” single-particles and cells, as biological data. We also can “read” them for further single-particle analysis.

The initially introduced magnetophoretic circuits operate in a two-dimensional (2D) in-plane magnetic field. But when two or more particles come into contact, we need to deal with the side effect of cluster formation. To overcome this problem, we added a vertical bias field and designed magnetophoretic circuits operating in a three-dimensional (3D) magnetic field^52–54^. The vertical bias field produces a repulsion force between the particles, prevents particle nucleation, and ensures operation at the single-particle level (i.e., transportation of individual magnetic micro-particles, not physically attached, in a particle population).

To have a complete circuit design suitable for transporting particles to any desired point in a plane, particle movement in both x- and y- directions and switching direction between them is needed. We have shown this operation in the circuits working in 2D magnetic fields; however, we have not introduced them in the drop-shape circuits operating in 3D fields, as the most efficient design^52^, yet. That is because bending the magnetic tracks results in large gaps between the “drop” sections in the magnetic pattern, which is problematic in transporting the magnetic particles from one magnet to another. Here, by introducing the magnetophoretic bends, we answer this need for manipulating particles in the size range of ~ 2.8–20 µm. This achievement is crucially important because it allows full device operation while it overcomes the cluster formation problem.

Since the device operates at the single-particle level, it opens the window for fundamental single-particle applications. Micromagnets have previously been used to detect bioparticles^55–57^. In this method, an array of micromagnets is exposed to an in-plane rotating field, and cluster formation of ligand-functionalized magnetic beads on the chip indicates the capture of the analyte of interest in between them. But, (1) in a rotating in-plane magnetic field, even without binding via analytes, the particles may form agglomerates. (2) Also, the proposed method is based on the detection of the average optical intensity of the reflected light from the magnetic particles on the chip. Thus, it is an average-based detection method.

One application of the proposed chip in this study is to detect the biological analytes like the one mentioned above while it answers its limitations. (1) The vertical bias field in the 3D magnetic field used in the current work prevents particle cluster formation when no analyte exists. In the Results and Discussions section, we show that the average number of pairs in this condition (i.e., in a 3D magnetic field) dropped more than 7 times. The unwanted pair formation in the 2D fields is considered as a background noise which makes predicting low-level analytes (i.e., analyte concentrations less than ~ 10^–12^) challenging. (2) Moreover, our proposed tool operates at single-particle resolution. We can detect single particle pairs using our image MATLAB processing code. Hence, it allows detection of ultimately low levels of analytes (concentration of ~ 10^–13^) captured in between only two beads (i.e., enhanced sensitivity). (3) Furthermore, it allows the detection of multiple analytes in a single chip. We have seen normal chip operation within a month after fabrication. Also, the chips can be reused after rinsing with de-ionized water.

## Results and discussions

The applications of our tool can be divided into two main categories of “particle transport” and “analyte detection”. Hence, we divide this section into two sub-sections. In the first one, we mention the underlying equations governing particle transport. We explain why the circular pattern is problematic and how we overcome the challenge by applying a vertical bias field and using the drop-shape design. Then, we discuss the ability of the bent magnetic tracks in transporting magnetic particles of different sizes and come up with design rules. We provide both simulation and experimental results for various designs. The results in this sub-section are sufficient to design a circuit to accurately transport single-particles to desired spots on the chip.

Then, in the next sub-section, we present the experimental results for detecting analytes of interest, using the proposed tool. We show the ability of the device in detecting proteins and DNA fragments, captured by the ligand-functionalized magnetic beads. We compare the results obtained in a 3D magnetic field and show the improvement achieved compared to the results achieved when using the 2D field configuration.

### Magnetophoretic circuit design

To appropriately design the magnetophoretic circuits, the relation between the magnetic potential energy and the magnetic force on a magnetic particle exposed to a magnetic field needs to be considered as stated in Eq. (1)^58^:

1 $$U = - \mathop \int \limits_{{r_{i - 1} }}^{{r_{i} }} \vec{F}.\vec{d}r$$

where F and U are the magnetic force for transporting the particle from point r_i-1_ to r_i_ and magnetic potential energy difference between the two points, respectively. The magnetic energy in Eq. (1) can be calculated as shown in Eq. (2)^58^:

2 $$U = \frac{1}{2}\mu_{0} V_{p} \left( {{\upchi }_{p} - {\upchi }_{f} } \right)H^{2}$$

where μ_0_, V_p_, χ_p_, χ_f_, and H stand for vacuum magnetic permeability, particle volume, particle magnetic susceptibility, fluid magnetic susceptibility, and magnetic field intensity, respectively. Then, the velocity for a spherical magnetic particle in aqueous fluids (which is the case in our study), using an overdamped first-order motion equation, can be written as Eq. (3)^59^:

3 $$v = \frac{F}{{6\pi_{f} R_{p} }}$$

where η_f_ and Rp represent the fluid viscosity and the particle radius. In order to predict the trajectory of the particle, it can be considered as a series of short straight paths (with laminar flow only) and a simple forward difference scheme, where $r_{i} = r_{i - 1} + v_{i - 1} \Delta t$ defines the particle position based on its previous position and velocity, can be used^46^. The small friction (including rotational friction) forces can be neglected. Since our chips are covered with non-fouling layers, the chance of particles-surface bond formation is too low. Moreover, since the particles move around the magnets with a trajectory outside the magnetic pattern, we do not need to consider the dynamics of particles moving over the 100 nm thick magnetic thin film. Thus, based on Eq. (1), the magnetic particle exposed to a magnetic field gradient moves towards the area with minimum dipolar energy, and by studying energy distribution the particle trajectories can be predicted. We used Eq. (2) in COMSOL software to simulate the energy distribution (See “Materials and methods” section).

The locations of the dipolar energy minima in linear magnetizable systems are at points where the outward normal component of the magnetic pattern curvature is parallel to the external magnetic field. Thus, in the magnetic disks used in magnetophoretic circuits operating in 2D in-plane magnetic fields, two energy wells form on their opposite sides (i.e., the north and south poles). Magnetic particles and cells tend to move to these energy wells. In a rotating magnetic field, they periodically switch between the north and south poles and move along the magnetic tracks composed of connected magnetic disks^49^.

**Figure 1:**
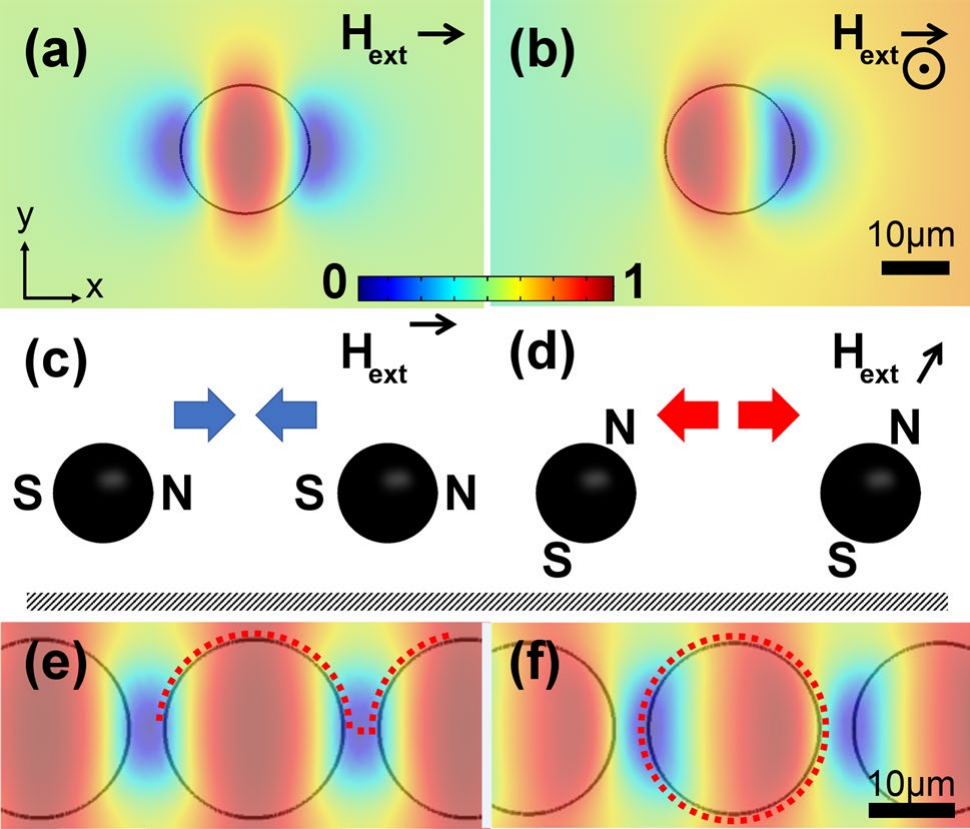
The effect of vertical bias field in magnetophoretic circuits. The magnetic energy landscape simulation results above the substrate for a magnetic disk, (**a**) exposed to an in-plane magnetic field, and (**b**) after superimposing a vertical bias field are shown. The blue and red areas stand for the regions with low and high magnetic energies, respectively. Here, we did a min–max normalization to scale the magnetic energy between 0 and 1 (see the legend). The black arrow and the dot depict the external magnetic field direction and the vertical magnetic fields, respectively. Side view schematics of the magnetic particle alignment in an (**c**) in-plane magnetic field and (**d**) after superimposing a vertical bias field are illustrated. The black circles and the shaded area depict the magnetic particles and the substrate, respectively. The blue and red arrows stand for the attractive and repulsive forces, respectively. N and S depict the north and south poles, respectively. The magnetic energy landscape simulation results above the substrate for a disk-based magnetophoretic circuit (**e**) exposed to an in-plane magnetic field and (**f**) after superimposing a vertical bias field are illustrated. The dotted lines stand for the particle trajectories.

The fundamental problem of manipulating single particles in magnetophoretic circuits operating in a 2D field comes from the micro-disk rotational symmetry in the in-plane rotating magnetic fields, where the dipoles cannot identify the north and south poles of the magnetic thin film patterns. Superimposing a vertical field to the in-plane rotating field (1) turns one of the energy wells (i.e., attractive poles) into an energy hill (i.e., repulsive pole) (See Fig. 1a,b for the simulation results, where blue and red area depict the regions with low and high magnetic energy) and (2) provides a repulsion force between the particles (See Fig. 1c,d). As shown in Fig. 1c (side view), in an in-plane magnetic field the opposite poles of the two adjacent magnetic particles meet and attract each other. However, as illustrated in Fig. 1d, when particles are biased, the force between them is repulsive. These attractive and repulsive forces between the two particles, as two magnetic point dipoles, can be described as Eq. (4)^60,61^:

4 $$F = \frac{{3\mu_{0} }}{{4\pi r^{5} }}\left[ {\left( {m_{1} .r} \right)m_{2} + \left( {m_{2} .r} \right)m_{1} + \left( {m_{1} .m_{2} } \right)r - \frac{{5\left( {m_{1} .r} \right)\left( {m_{2} .r} \right)}}{{r^{2} }}r} \right]$$

where $m_{i} = \frac{{4\pi R_{p}^{3} {\upchi }_{p} H}}{3}$ is the particle dipole moment and r stands for the distance between the two particles. In the cases in which the vertical fields are stronger than the in-plane field, the force in Eq. (4) becomes negative, depicting the repulsive force between the particles. The more complicated force calculation can be found elsewhere^62^. Although we did not carefully study whether the particles can overcome the downward gravity force and the downward magnetic force applied to the particle from the magnetic thin film, be levitated, and form vertical agglomerates, we did not see them being formed in our experiments.

**Figure 2:**
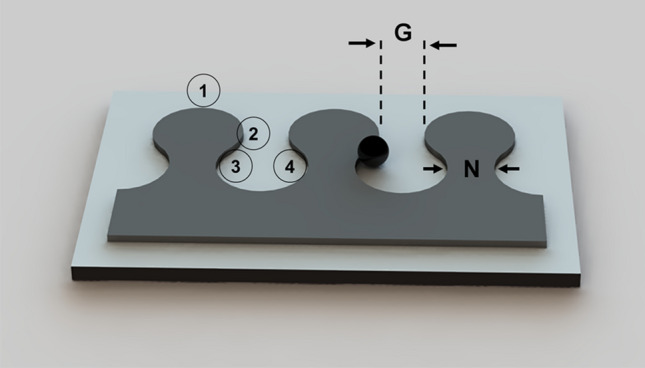
Schematic of the drop-shape magnetophoretic circuit design. The black circle depicts a sample particle, and the numbers stand for the sequence of the particle positions when it moves along the magnetic track. In our fabricated chips, G is 15 µm.

In disk-based magnetophoretic circuits operating in a 2D in-plane magnetic field, as shown in Fig. 1e, the particles switch between the poles of adjacent magnetic disks. But, in the presence of the bias field and a rotating magnetic field, as illustrated in Fig. 1f, the particles move in a closed loop around a single disk and cannot move along the magnetic track (See the red dotted line in Fig. 1e,f for the particle trajectory in each case). As seen in Fig. 1f, since the pole at the adjacent disk shows an energy hill, the problem cannot be answered by adjusting parameters such as disk diameter or gap size. To overcome this challenge, we design magnetic patterns consisting of alternating sections of positive and negative curvatures so that particle transport in a 3D magnetic field is possible. The result is the drop-shape magnetic patterns, shown in Fig. 2, where particles move along the magnetic track^52^. In this figure, the numbers stand for the sequence of the particle positions when it moves along the magnetic track from one magnet (the “drop” section of the track) to another.

Figure 2 shows the transport mechanism in a 3D magnetic field on the drop-shape magnetic track which involves smooth transports along the sections with positive curvatures (e.g., from point 1 to point 3 in Fig. 2), followed by sudden transitions at sections with negative curvatures (e.g., from point 3 to point 4). We introduce β = d_P_/G and γ = d_P_/N as two dimensionless parameters, where d_P_, G, and N stand for the particle diameter, the pattern gap, and the pattern neck size (See Fig. 2), respectively. In a straight magnetic track, similar to the one in Fig. 2, in our studies, we see appropriate particle transport for β > 0.14 and γ < 1.4.

**Figure 3:**
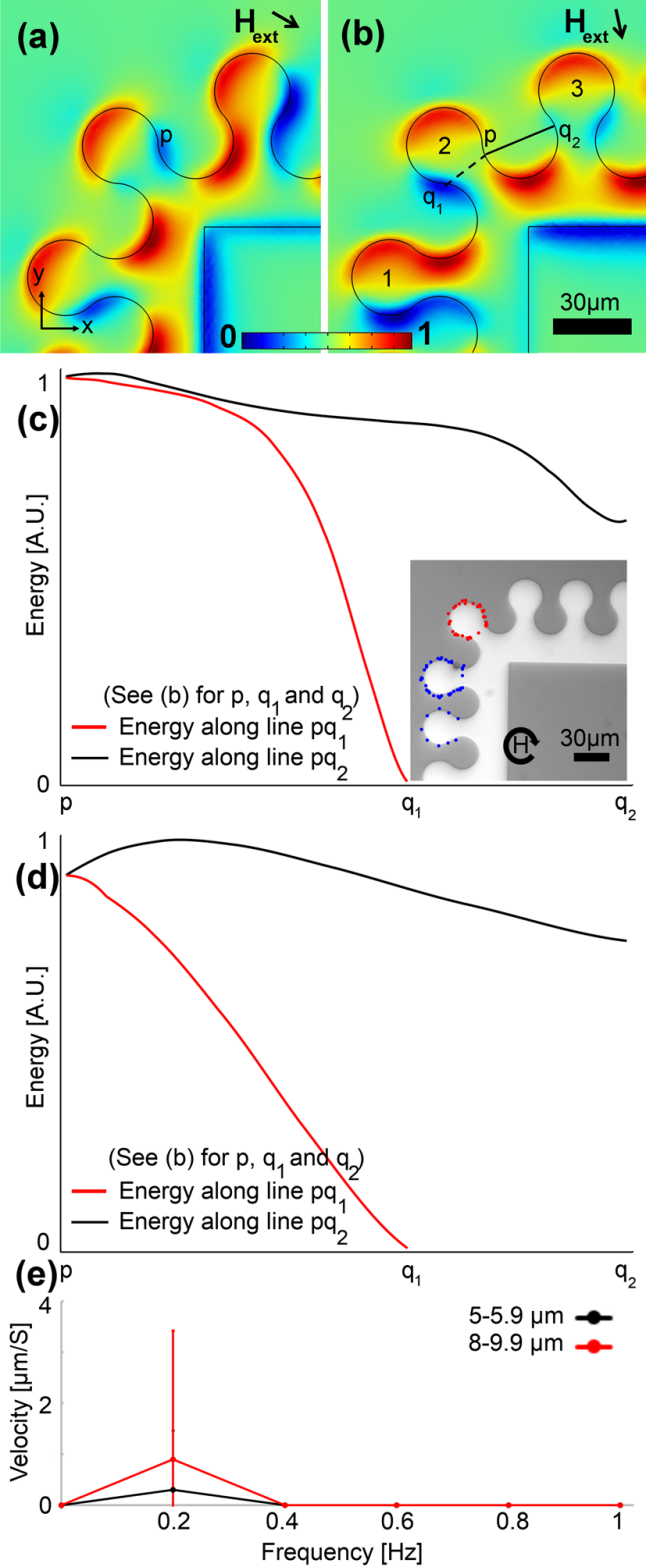
A sample inappropriate magnetophoretic bend design. (**a**,**b**) The magnetic energy landscape simulation results above the substrate for a sample bend are illustrated. The blue and red areas stand for the regions with low and high magnetic energies, respectively. The black arrow depicts the in-plane magnetic field direction, in addition to which a vertical bias field is applied. Here, we did a min–max normalization to scale the magnetic energy between 0 and 1 (see the legend). The magnetic energies along the pq_1_ and pq_2_ lines in (**b**) (i.e., the dashed and solid lines) are plotted with the red and black curves for particles with a diameter of (**c**) 5 µm and (**d**) 20 µm. The inset illustrates the experimental trajectories for two sample particles at a frequency of 0.2 Hz with blue and red dotted lines, respectively. The circular arrow in the inset depicts the magnetic field rotation. The goal is to transport the particles from magnet 1 to magnet 3 through magnet 2. We used a linear scale for the magnetic energy plots and we did a min–max normalization, using the minimum and maximum of the magnetic energy along the pq_1_ and pq_2_ paths, to scale the energy between 0 and 1. (**e**) The particle velocity versus frequency of the externally applied magnetic field is plotted with the black and red curves for two particle sets with sizes in the ranges of 5–5.9 µm and 8–9.9 µm, respectively. The error bars show standard deviations.

To switch the particle transport direction between x- and y- directions, obtuse and acute bends are needed. We designed and tested several obtuse bends, an example of which is illustrated in Fig. 3. These patterns are designed by enlarging the gap between the magnet numbers 1 and 2 (and 2 and 3), which negatively affects β (i.e., they cannot transfer small particles). The goal here is to transport the particle from magnet 1 to magnet 3 through magnet 2. The magnetic energy distribution in Fig. 3a shows the blue areas at which particles initially stay (e.g., point p). By rotating the magnetic field in Fig. 3b, the particle at point p has two paths to choose to the blue areas of q_1_ or q_2_. By plotting the magnetic energy along lines pq_1_ and pq_2_ at heights of 2.5 µm and 10 µm in Fig. 3c,d, we realize that an energy barrier along the pq_2_ path exists. Thus, the particle chooses the pq1 path and cannot move from magnet number 2 to magnet number 3. Our experimental results agree with these simulation predictions. In the inset of Fig. 3c, we show the experimental trajectories of two sample particles with blue and red dotted lines, respectively, circulating the magnets. The particle with the red trajectory in this figure has been placed on the magnet on the corner initially, and it cannot move to the next magnet on the track. The particle with the blue trajectory in this figure moves from one magnet to the next one in the area with an appropriate geometry (i.e., straight track). But, in the next step, due to an inappropriate β on the corner, it cannot move to the magnet there. Thus, this design is not appropriate for transporting magnetic particles. Figure 3e shows the experimentally measured particle velocity at various external field frequencies. Only rare particles (2 out of 15) randomly could jump forward at low frequencies, which cannot be considered proper particle transportation, resulting in larger error bars (standard deviation) compared to the results at higher frequencies. This phenomenon may be due to random particle parameters or defects. At high frequencies, we did not observe any jump between the magnets in the experiment, which may be because the particles do not have enough time to follow the energy wells that move fast (because of the large gap between the magnets in this geometry).

**Figure 4:**
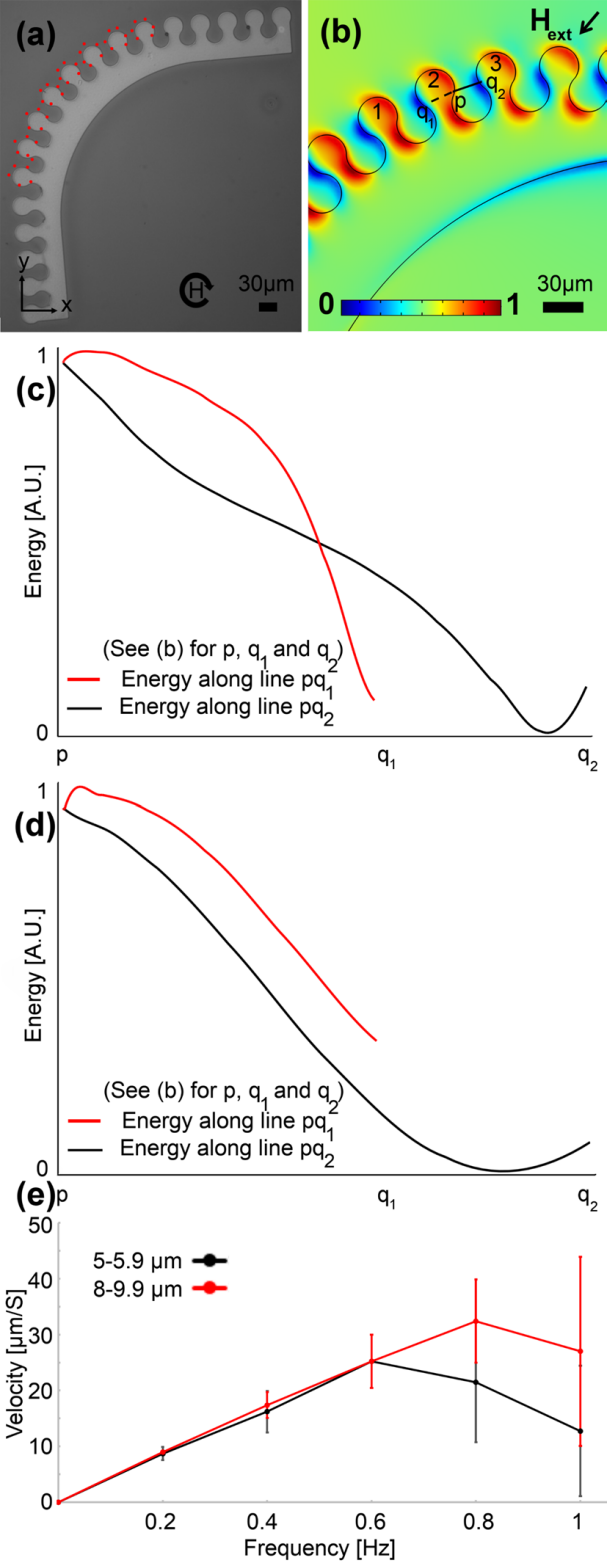
A sample appropriate magnetophoretic bend design. (**a**) The experimental image is shown, where the red dotted line stands for the particle trajectory at a frequency of 0.2 Hz. The circular arrow depicts the magnetic field rotation. (**b**) The magnetic energy landscape simulation result above the substrate is illustrated. The blue and red areas stand for the regions with low and high magnetic energies, respectively. Here, we did a min–max normalization to scale the magnetic energy between 0 and 1 (see the legend). The black arrow depicts the in-plane magnetic field direction, in addition to which a vertical bias field is applied. The magnetic energies along the pq_1_ and pq_2_ lines in (**b**) (i.e., the dashed and solid lines) are plotted with the red and black curves for particles with a diameter of (**c**) 5 µm and (**d**) 20 µm. The goal is to transport the particles from magnet 1 to magnet 3 via magnet 2. We used a linear scale for the magnetic energy plots and we did a min–max normalization, using the minimum and maximum of the magnetic energy along the pq_1_ and pq_2_ paths, to scale the energy between 0 and 1. (**e**) The particle velocity versus frequency of the externally applied magnetic field is plotted with the black and red curves for two particle sets with sizes in the ranges of 5–5.9 µm and 8–9.9 µm, respectively. The error bars show standard deviations.

To overcome the challenge in the design shown in Fig. 3, we propose several other bend designs, in which we keep β and γ in the appropriate range. The first design in which the gap (G = 15 µm), and thus β, are kept very close to the one in the original straight tracks (i.e., the one shown in Fig. 2) is illustrated in Fig. 4a. In this design, the corner is replaced with many magnets each of which has a slight angle with respect to the main magnetic track. The energy simulation result for this design at the time of particle switching from one magnet to another is presented in Fig. 4b. The energy along paths pq_1_ (backward path) and pq_2_ (forward path) are shown in Fig. 4c,d, with the red and black curves, respectively. Figure 4c,d stand for the energies at the center of particles with diameters of 5 and 20 µm, respectively. The peaks in the red curves and the negative slopes in the black curves show that the particle moves forward along the magnetic track from one magnet to the next one. Our experiments show that the particle transport in this design is smooth (See red dotted lines in Fig. 4a for the experimental particle trajectories). We repeated these experiments at various frequencies (See Fig. 4e), and we found that the particles with different sizes (5–9.9) can move well at frequencies below ~ 0.6 Hz. But, the drawback of this design is the relatively large occupied space, due to the number of drop-shape magnets, this bend needs.

**Figure 5:**
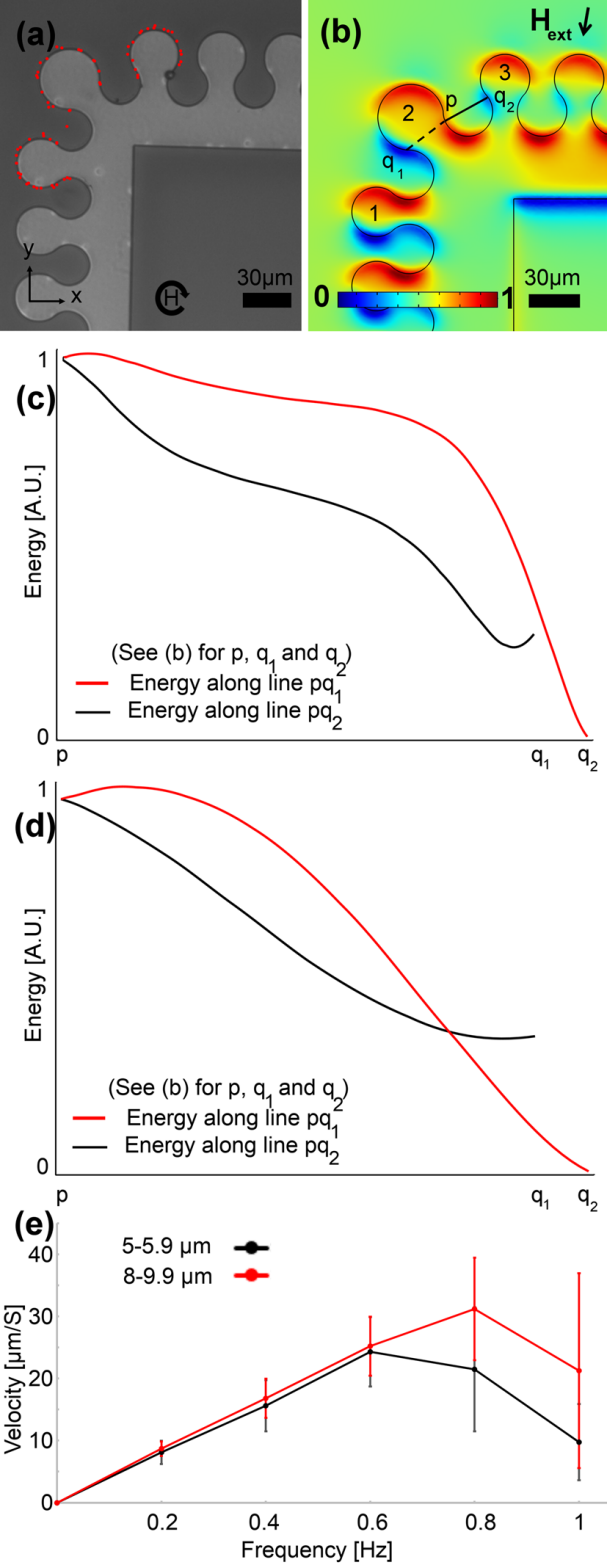
A sample appropriate magnetophoretic bend design. (**a**) The experimental image is shown, where the red dotted line stands for the particle trajectory. The circular arrow depicts the magnetic field rotation. (**b**) The magnetic energy landscape simulation result above the substrate is illustrated. The blue and red areas stand for the regions with low and high magnetic energies, respectively. Here, we did a min–max normalization to scale the magnetic energy between 0 and 1 (see the legend). The black arrow depicts the in-plane magnetic field direction, in addition to which a vertical bias field is applied. The magnetic energies along the pq_1_ and pq_2_ lines in (**b**) (i.e., the dashed and solid lines) are plotted with the red and black curves for a particle with a diameter of (**c**) 5 µm and (**d**) 20 µm. The goal is to transport the particles from magnet 1 to magnet 3 via magnet 2. We used a linear scale for the magnetic energy plots and we did a min–max normalization, using the minimum and maximum of the magnetic energy along the pq_1_ and pq_2_ paths, to scale the energy between 0 and 1. (**e**) The particle velocity versus frequency of the externally applied magnetic field is plotted with the black and red curves for two particle sets with sizes in the ranges of 5–5.9 µm and 8–9.9 µm, respectively. The error bars show standard deviations.

**Figure 6:**
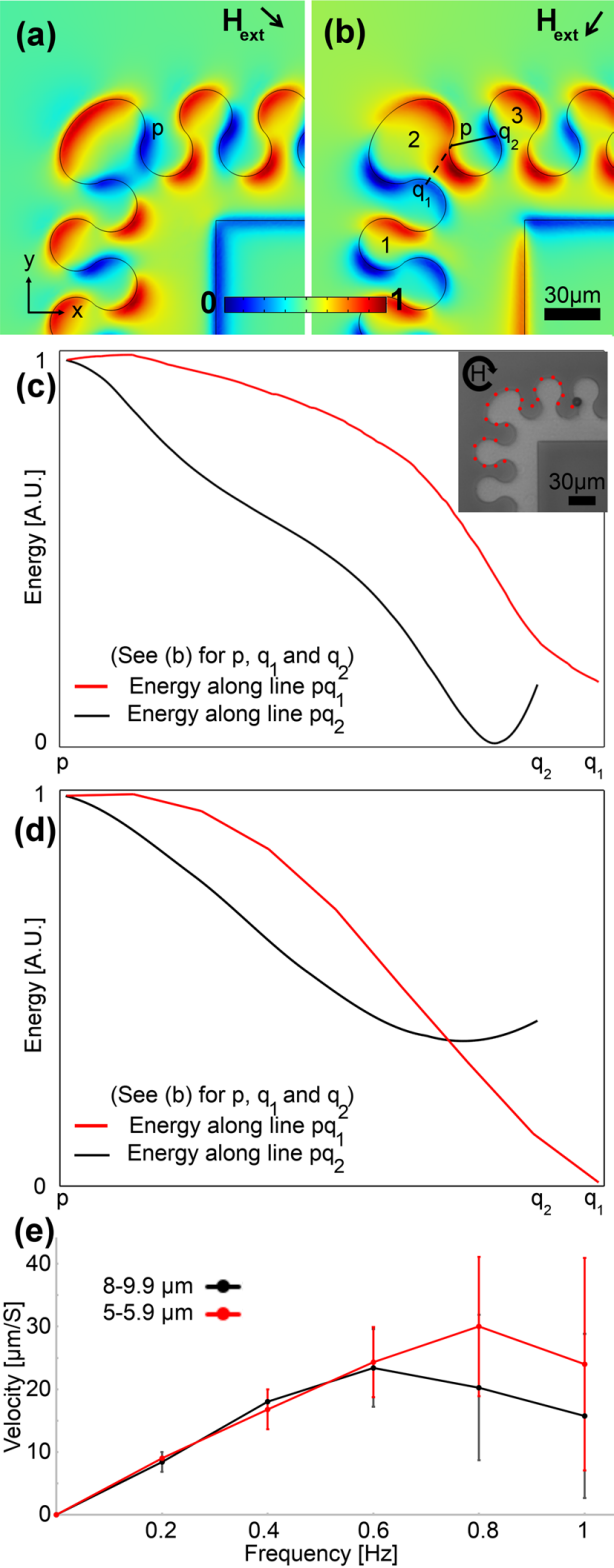
A sample appropriate magnetophoretic bend design. (**a**,**b**) The magnetic energy landscape simulation results above the substrate are illustrated. The blue and red regions stand for the regions with low and high magnetic energies, respectively. Here, we did a min–max normalization to scale the magnetic energy between 0 and 1 (see the legend). The black arrow depicts the in-plane magnetic field direction, in addition to which a vertical bias field is applied. The magnetic energies along the pq_1_ and pq_2_ lines in (**b**) (i.e., the dashed and solid lines) are plotted with the red and black curves for a particle with a diameter of (**c**) 5 µm and (**d**) 20 µm. The red dotted line in the inset in (**c**) illustrates a sample particle trajectory, where the circular arrow depicts the magnetic field rotation. The goal is to transport the particles from magnet 1 to magnet 3 via magnet 2. We used a linear scale for the magnetic energy plots and we did a min–max normalization, using the minimum and maximum of the magnetic energy along the pq_1_ and pq_2_ paths, to scale the energy between 0 and 1. (**e**) The particle velocity versus frequency of the externally applied magnetic field is plotted with the black and red curves for two particle sets with sizes in the ranges of 5–5.9 µm and 8–9.9 µm, respectively. The error bars show standard deviations.

To enhance the bend design, we came up with the patterns shown in Figs. 5 and 6, where, as opposed to employing several drop-shape magnets, we use a single large magnet on the corner, which results in an appropriate β. The appropriate experimental trajectory shown in Fig. 5a is the result of the suitable energy distribution in Fig. 5b and is supported by the curves in Fig. 5c,d. Based on these curves the particle sees an energy barrier along the backward path (i.e., the red curves); however, it sees a negative energy slope along the forward path. Similarly, the energy distributions shown in Fig. 6a,b and the curves in Fig. 6c,d show that after reaching point p in Fig. 6a the particle moves to point q_2_ in Fig. 6b. This behavior is seen in both particle sets with diameters of 5 and 20 µm. The experimental particle trajectories illustrated in Fig. 5a and the inset of Fig. 6c, confirm our theoretical findings. Our experimental results show that both designs work well in transporting particles of various sizes (5–9.9) at frequencies below ~ 0.6 Hz (See Figs. 5e, 6e).

**Figure 7:**
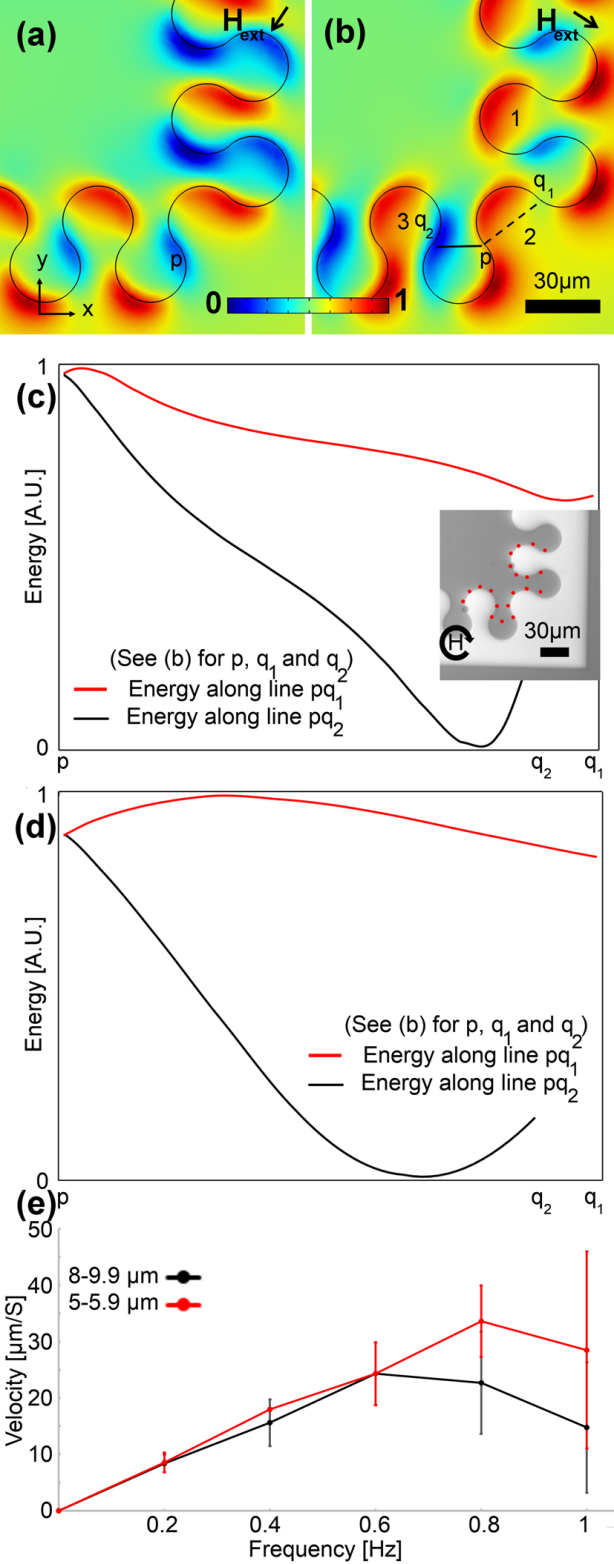
A sample appropriate magnetophoretic bend design for transporting particles inside the bend. (**a**,**b**) The magnetic energy landscape simulation results above the substrate are illustrated. The blue and red areas stand for the regions with low and high magnetic energies, respectively. Here, we did a min–max normalization to scale the magnetic energy between 0 and 1 (see the legend). The black arrow depicts the in-plane magnetic field direction, in addition to which a vertical bias field is applied. The magnetic energies along the pq_1_ and pq_2_ lines in (**b**) (i.e., the dashed and solid lines) are plotted with the red and black curves for a particle with a diameter of (**c**) 5 µm and (**d**) 20 µm. The red dotted line in the inset in (**c**) illustrates a sample particle trajectory, where the circular arrow depicts the magnetic field rotation. The goal is to transport the particles from magnet 1 to magnet 3 via magnet 2. We used a linear scale for the magnetic energy plots and we did a min–max normalization, using the minimum and maximum of the magnetic energy along the pq_1_ and pq_2_ paths, to scale the energy between 0 and 1. (**e**) The particle velocity versus frequency of the externally applied magnetic field is plotted with the black and red curves for two particle sets with sizes in the ranges of 5–5.9 µm and 8–9.9 µm, respectively. The error bars show standard deviations.

Sometimes we need to transport the particles inside the bend (see Fig. 7). As shown in Fig. 7a,b the particle at point p in Fig. 7a reaches point q2 in Fig. 7b. The curves in Fig. 7c,d show that this design works fine for particle sizes in the range of 5 to 20 µm (i.e., the particles see an energy barrier in the backward path while they see an energy well in the forward path). A sample experimental particle trajectory on this bend design is shown in the inset of Fig. 7c. Again, we saw appropriate particle transport at frequencies below 0.6 Hz for the particle size range of 5–9.9 Hz.

The particle transport speed depends on the applied external magnetic field frequency. As shown in Figs. 4, 5, 6 and 7, the particles with the diameter range of 5–5.9 µm reach the average speed of ~ 25.2 µm/s at the frequency of 0.6, after which the particle cannot follow the external field (because of the big drag force compared to the driving magnetic force) and the velocity drops. The particles with the size range of 8–9.9 µm, because of higher magnetic susceptibilities can move at the frequency of 0.8 Hz and reach the average speed of ~ 32.4 µm/s. Since we need complete stable conditions for our particle transports and sensing tests, we chose the frequency of 0.2 Hz, with an average speed of ~ 9 µm/s, for our next experiments. We also tested the movement of small (e.g., 2.8 µm) and large 14–17.9 µm particles at 0.2 Hz and observed smooth transport on the proposed magnetophoretic circuits. Also, we ran the simulations in the range of 2–20 µm, but we chose to show the results of 5 µm and 10 µm beads, as two examples.

**Figure 8:**
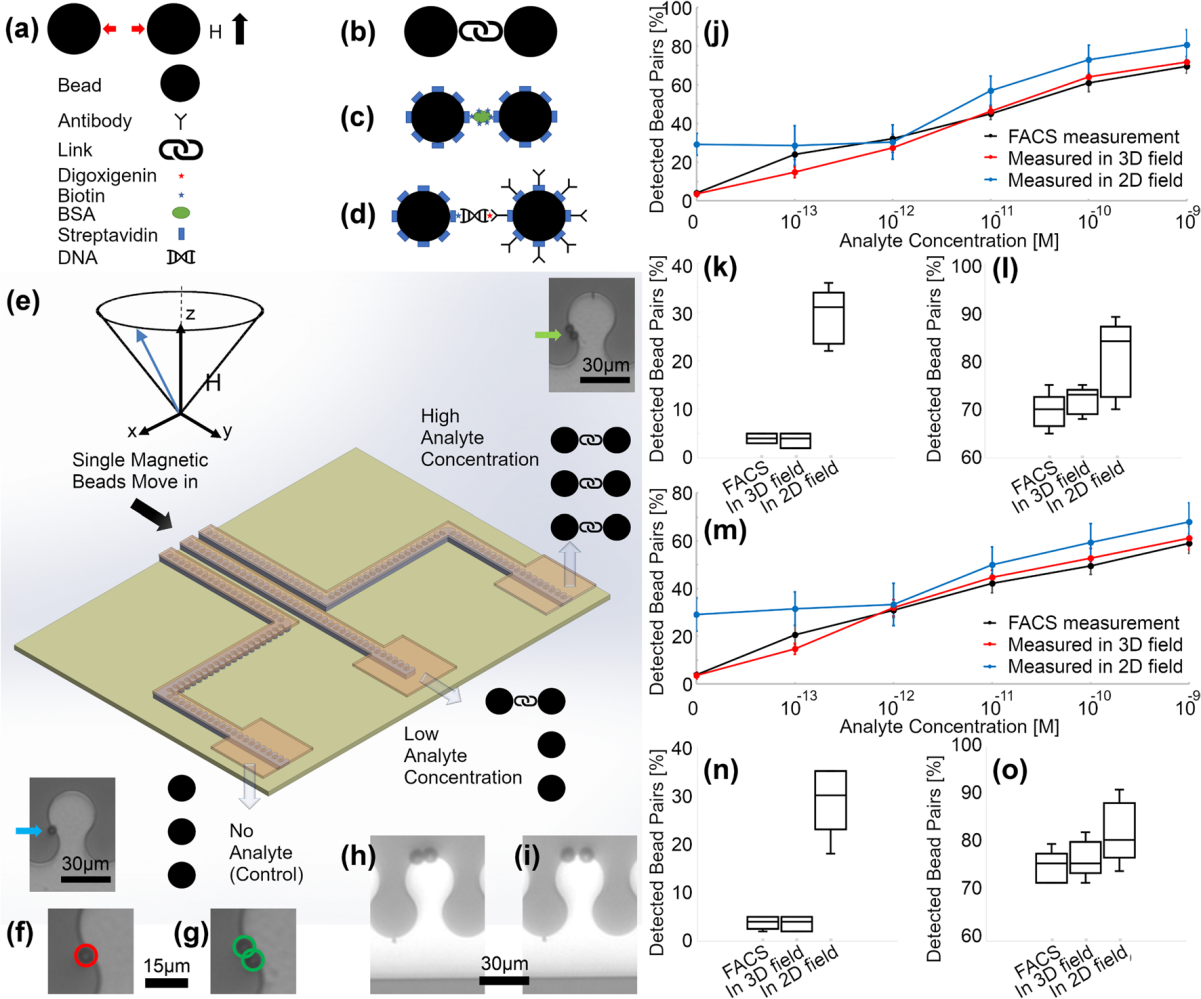
Schematic of the detecting magnetic bead pair formation. (**a**) The vertical bias field (shown by the black arrow) results in a repulsion force between the beads (depicted by the red arrows). (**b**) A link between the two beads forms a bead pair. This technique is suitable for the detection of analytes such as (**c**) proteins or (**d**) DNA fragments. (**e**) Schematic of the chip for transporting the detecting magnetic beads to the microchambers with various analyte concentrations. H shows the applied conical field. The insets on the top right and down left corners show a bead pair formed in high analyte concentration and a single bead when no analyte exists, respectively. The image processing code detects (**f**) single beads and (**g**) the bead pairs. (**h**) The unbonded particle pairs (**i**) are separated by the vertical bias field. (**j**) Bead pair percentage as a function of the BSA concentrations, based on the magnetophoretic circuits operating in a 3D magnetic field, magnetophoretic circuits operating in a 2D magnetic field, and FACS are plotted in red, blue, and black curves, respectively. (**k,l**) The box plots for the FACS, magnetophoretic circuits operating in a 3D magnetic field, and that operating in a 2D magnetic field at BSA concentration of 0 and 10^–9^, respectively, are shown. (**m**) Bead pair percentage as a function of the HSV DNA concentrations, based on the magnetophoretic circuits operating in a 3D magnetic field, magnetophoretic circuits operating in a 2D magnetic field, and flow cytometry are plotted in red, blue, and black curves, respectively. (**n,o**) The box plots for the FACS, magnetophoretic circuits operating in a 3D magnetic field, and that operating in a 2D magnetic field at a DNA concentration of 0 and 10^–9^, respectively, are illustrated.

The combination of the introduced patterns provides the opportunity to design magnetophoretic circuits for the precise transportation of magnetic particles. Towards this goal, we designed a circuit to transport magnetic particles to spots on a chip with analytes of interest for detection purposes. Figure 8 illustrates a schematic of a sample circuit design.

### Analyte detection

Here, we use our proposed magnetophoretic circuit as a tool to detect herpes simplex virus type 1 (HSV-1, or oral herpes) after transporting the bioparticles on-chip using the proposed magnetic circuit designs, without any need for amplification. The bend designs introduced in Figs. 6, 7 and magnetic beads with sizes of 2.8 µm (Dynabeads M-280), 6 µm (Bangs Laboratories 6 µm COMPEL) and 8 µm (Bangs Laboratories 6 µm COMPEL), resulting in $\beta ,\gamma \approx 0.18$; $\beta ,\gamma \approx 0.4$; and $\beta ,\gamma \approx 0.53$, respectively, were used. HSV is a DNA virus that belongs to the Herpesviridae family and affects more than 60% of the global population. Currently, culture-based or polymerase chain reaction-based methods are used for HSV diagnosis; however, more advanced sensitive and rapid HSV diagnostic tools are needed^63^. Here, we use our proposed chip to run a single-molecule detection assay based on the reaction of the analyte of interest (e.g., HSV UL27 gene) in between two magnetic beads. Similar to recent works^55–57^, in this method, we label the DNA fragments with biotin and digoxigenin oligonucleotide probes so that they can bind in between streptavidin and anti-digoxigenin antibody labeled magnetic beads, forming a magnetic bead pair/cluster (See Fig. 8), but, importantly, in a 3D magnetic field. To show the ability of the chip to detect multiple analytes simultaneously, we also use the chip to detect biotinylated bovine serum albumin (BSA), too. This detection is done via binding in between streptavidin-coated magnetic beads (See Fig. 8).

In a pure horizontal field (such as the fields in previous works), as shown in Fig. 1c, the magnetic beads attract each other and they may come in contact, even in the absence of the analyte of interest. This phenomenon may cause problems in demonstrating the real number of bead pairs linked with the analytes of interest. To overcome this challenge, in this work for the first time we use a vertical bias field, which intrinsically exists in our tool. In this magnetic field, in the absence of the analyte, the beads repel each other and do not come into contact (See Fig. 8a, where the red arrows depict the repulsion force between the beads). Thus, it lowers the chance of unwanted bead pair formation. But providing a link between the beads (See Fig. 8b) keeps them in contact. This link, as mentioned above, may be an analyte of interest such as a protein (See Fig. 8c) or a DNA fragment (See Fig. 8d).

Using the proposed magnetophoretic circuits in this work, we designed magnetic paths for transporting single magnetic beads to the spots (i.e., microchambers) with different analytes and various concentrations (See the schematic in Fig. 8e). After bead transportation, we first turn off the vertical bias field to let the beads meet, and then by turning it on, the beads with no link are separated, leaving only the detecting bead pairs (See Fig. 8h,j). We show that the number of bead pairs, formed in the microchambers from the loaded single beads, varies with respect to the available analyte concentration in each microchamber on the chip. Examples of a detected single bead and bead pair in a chamber with no analyte and high analyte concentrations, respectively, are shown in the insets of Fig. 8e as well as Fig. 8f,g where we use a simple image processing Matlab code to detect them. The experimental results based on the proposed method for the protein of interest and the DNA of interest are presented in Fig. 8j,m, respectively. These plots, as well as the box plots in Fig. 8k,l,n,o, show the close agreement between the results based on our proposed method (i.e., based on circuits operating in a 3D field) and FACS (Fluorescence-Activated Cell Sorting). But these plots depict that the device operating in the 2D field shows a larger number of pairs at low concentrations (< 10^–12^) which makes analyte detection at these levels problematic (See flat curves in Fig. 8j,m at these concentrations). At high concentrations, the median and the error bars in the 2D field are larger than the ones based on the 3D field. These results confirm that our magnetophoretic circuits proposed in the current work operate as a more accurate biosensor.

Although it is out of the scope of the current work, detecting particle pairs after formation can also be transported. The trajectory of the particle pairs is similar to that of the single particles; however, since the drag force varies and, in addition to moving along the magnetic tracks, they experience a torque synced with the external magnetic field, the observed dynamic is more complicated.

## Materials and methods

**Figure 9:**
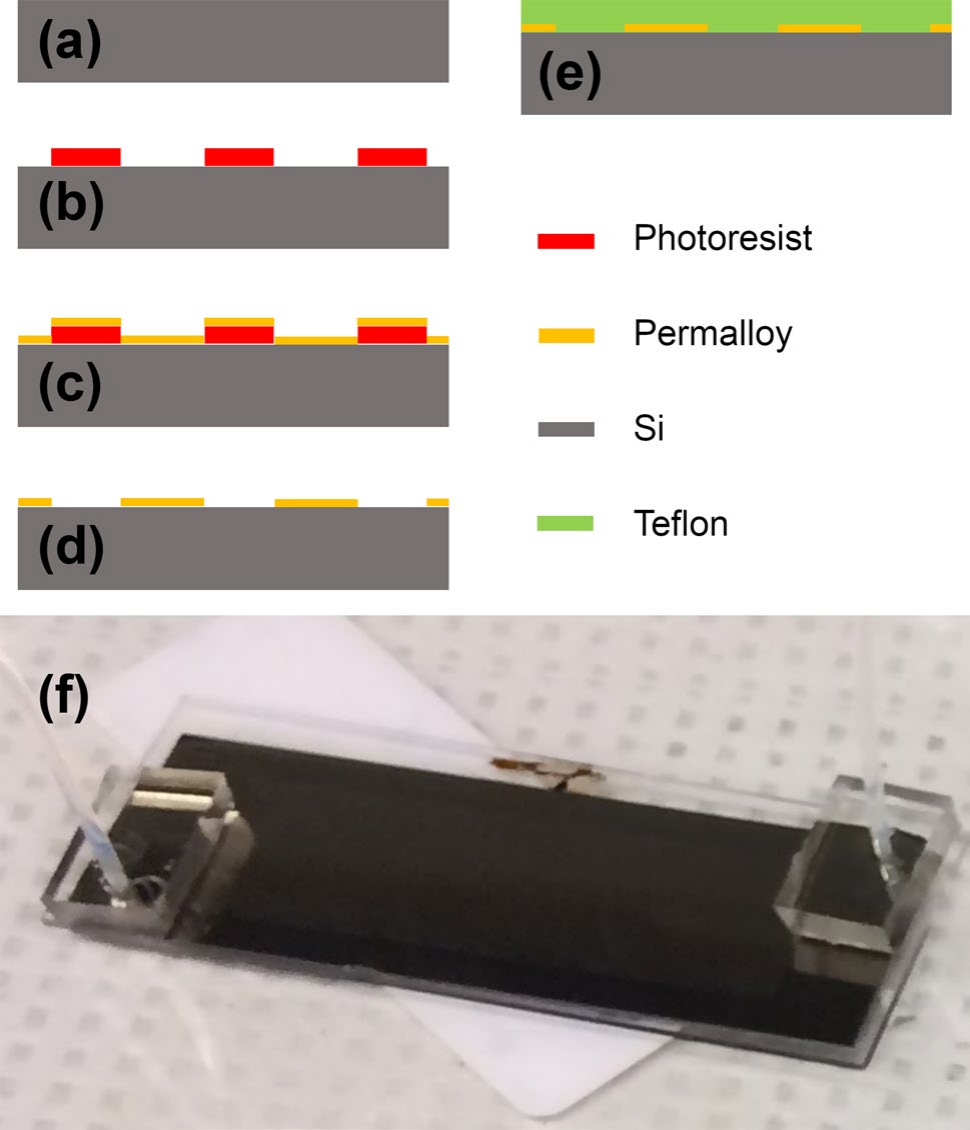
Microchip fabrication protocol. (**a**) Starting from a silicon wafer, (b) photoresist is patterned, (**c**) the chip is covered by permalloy, (d) liftoff removes the excess material, and (**e**) the chip is covered by a non-fouling layer (e.g., Teflon). (**f**) A sample complete microfluidic chip is shown.

The microfabrication steps are explained elsewhere^50^. In short, silicon wafers (University Wafer, Boston, MA, USA) were rinsed with acetone and then with isopropanol. The chips were dried with nitrogen gas. A photoresist NFR16-D2 (JSR Micro Inc., Sunnyvale, CA) layer was made on the chips by spin-coating for 5 s at 500RPM, and then for 30 s at 3000RPM. Next, the chips were soft-baked at 90 °C for 2 min on a hotplate. Then, the wafers were exposed to ultraviolet light for 12 s at an illumination power of 13.5 mW at a wavelength of 365 nm (Karl Suss MA6/BA6). Then, they were hard-baked at 90 °C for two minutes. Next, after keeping the chips in developer solution (i.e., Microposit MF-319, Shipley, Marlborough, MA) for a minute, they were rinsed with deionized water and dried with nitrogen gas. A 5 nm/100 nm stack of Ti/Ni80Fe20 film on the chips was fabricated using electron-beam evaporation (Kurt Lesker PVD 75), at the operating pressure of 1 × 10^−5^. Next, 1165 resist remover (NMP) was used for the lift-off process and removing the extra metal. After rinsing with acetone and isopropanol, the chips were dried with nitrogen gas. Then, the chips were spin-coated with a non-fouling layer such as Teflon thin film to lower the particle to surface adhesion. See the schematics in Fig. 9a-e for the fabrication processes. In cases of having multiple chambers on the chip, they were etched in a glass slide which then was bonded on the chip using the anodic bonding technique (See Fig. 9f for an example of a fabricated chip). Other methods such as SU8 molding can be used too.

The experiments with the commercially available spherical magnetic beads made of iron oxide and polystyrene (Spherotech CM-50-10 (~ 5–5.9 µm, carboxyl)^64^, FCM-8056–2 (8.0–9.9 µm, carboxyl)^64^, Spherotech CM-150–10 (14.0–17.9 µm, carboxyl)^64^, Dynabeads M-280 Streptavidin (~ 2.8 µm)^65^), Bangs Laboratories UMC0101 6 µm COMPEL (Streptavidin^66^, and Bangs Laboratories UMC0102 8 µm COMPEL (Streptavidin)^66^) were performed in de-ionized water and phosphate buffered saline (PBS).

The rotating magnetic field was applied by a custom-designed four-pole magnetic structure wrapped with magnetic wire (20 AWG). The coils were powered by programmable power supplies (Kepco BOP 20-5) which were controlled by a customized LabVIEW program (National Instrument). To maximize the uniformity of the magnetic field exposed to the chip, we placed it in the middle of the magnetic coils. We experimentally got good results with a chip with a maximum size of ~ 7 cm (the size of a glass slide) in between coils with a distance of 12 cm. However, we suggest limiting the circuit design to a 2.5 × 2.5 cm area on chips to ensure the magnetic field uniformity, if the coils are not placed in place carefully. The vertical and horizontal components of the magnetic field were 70 Oe each. The magnetic field frequency in all sensing experiments was set to 0.2 Hz.

The simulations were run in COMSOL Multiphysics^®^ 5.3 software. First, the geometries of the models were created and placed inside a cubic box, resembling the surrounding environment. Then the study domain was chosen (Stationary) and the physical parameters and materials were defined. Permalloy 80 was chosen as the material for the magnetic patterns and water was chosen for the surrounding environment. Since we use COMSOL to only model the magnetic energy distribution, the only important parameter is the magnetic susceptibility of the materials. The appropriate mesh was chosen (maximum element size: 0.2 μm, minimum element size: 0.02 μm) to ensure convergent and accurate results. Then, the problem was solved by the Magnetic Field module. To produce the required magnetic field, it is possible to design coils and set the required electric current as the boundary conditions. But, to make the modeling easier, magnetic scalar potential can be set on the surrounding box faces. The values of the magnetic scalar potential are set such that the desired magnetic field is results near the magnets at the center of the box. Equations (5,6) are used in the COMSOL simulations:

5 $$H = - \nabla V_{m}$$

6 $$\nabla .B = 0$$

where V_m_ stands for the magnetic scalar potential. We plugged Eq. (2) into COMSOL software to calculate the resulting magnetic energy.

## Conclusions

Assembling enough number of single bioparticles in arrays on a chip is considered a promising method for dynamic phenotypic and genotypic single-particle studies. In this work, we proposed a hybrid full magnetophoretic circuit design operating in a 3D magnetic field to both transport single particles to specific spots in a plane and to detect analytes of interest. This achievement became possible by introducing bend designs to switch the particle trajectories in x- and y- directions. We ran computer simulations and experiments to systematically study the operation of the proposed bends with various particle sizes and at different operating frequencies. The vertical bias magnetic field in the proposed magnetophoretic circuits results in a repulsion force between the particles, which resembles the behavior of electrons in electrical circuits. This repulsion force lowers the chance of forming particle clusters which is important in single-particle analysis.

One important application of the proposed chip is in the field of bio-detection. We showed the ability of the proposed chip in detecting a model protein and a viral DNA. The analyte of interest provides a link between two particles and forms a particle pair. We showed that the analyte concentration is proportional to the number of particle pairs. The vertical bias field in this method plays a key role in lowering the chance of unwanted particle pair formation (i.e., in the absence of the analyte of interest) which normally happens in 2D in-plane fields. We showed that the particle-pair formation in the absence of analyte in 2D magnetic field is more than 7 times compared to the ones in 3D field and measurements based on FACS. Thus, by introducing the vertical magnetic field we could overcome this high noise level which makes prediction at low analyte concentrations (< 10^–12^) in systems operating in 2D field challenging. Also, in our proposed tool we saw standard deviations smaller than half of the one seen in the device operating in 2D field. The proposed chip not only enhances the detection specificity but also operates at single-particle resolution. It means it detects ultimately low levels of analytes captured in between only two beads, which is possible using an image processing Matlab code, as opposed to average-based optical bulk-level particle detection methods. Moreover, by adding multiple microchambers in the chip design, detection of multiple analytes on a single chip is possible. The proposed chip opens the window for designing flawless magnetophoretic circuits operating in a 3D magnetic field with applications in single-cell biology and medicine.
